# Novel deep intronic variants in *NTRK1* underlying congenital insensitivity to pain with anhidrosis

**DOI:** 10.3389/fgene.2026.1852317

**Published:** 2026-07-02

**Authors:** Xin Chen, Shuang Li, Zhe Liu, Jian Cheng, Xiuzhi Ren, Xiuli Zhao

**Affiliations:** 1 State Key Laboratory of Complex, Severe, and Rare Diseases, Center for Rare Diseases, Peking Union Medical College Hospital, Chinese Academy of Medical Sciences and Peking Union Medical College, Beijing, China; 2 State Key Laboratory for Complex, Severe, and Rare Diseases, Department of Medical Genetics, Institute of Basic Medical Sciences Chinese Academy of Medical Sciences, School of Basic Medicine Peking Union Medical College, Beijing, China; 3 Stem Cell Facility, National Infrastructures for Translational Medicine, Institute of Clinical Medicine, Peking Union Medical College Hospital, Chinese Academy of Medical Sciences and Peking Union Medical College, Beijing, China; 4 Children’s Hospital of Soochow University, Suzhou, China

**Keywords:** congenital insensitivity to pain with anhidrosis, deep intronic variants, *NTRK1*, pathogenic mechanisms, splicing

## Abstract

**Objectives:**

Congenital insensitivity to pain with anhidrosis (CIPA) is a rare autosomal recessive disorder caused by mutations in *NTRK1* that is characterized by pain insensitivity, anhidrosis, and recurrent fever. While genetic testing is the gold standard for CIPA diagnosis, the complexity of *NTRK1* variants poses major challenges. Conventional sequencing that is limited to the coding regions of *NTRK1* results in misdiagnoses or missed diagnoses in approximately 57% of patients. Accordingly, to improve the diagnostic efficiency of CIPA, we integrated whole-genome sequencing (WGS) with functional assays to identify deep intronic variants in *NTRK1*.

**Methods:**

All 18 probands were initially screened using polymerase chain reaction (PCR) and Sanger sequencing covering all exons and canonical splice sites of *NTRK1*. For patients with only one identified pathogenic allele, WGS was performed to detect potential deep intronic variants. Candidate variants were functionally validated using reverse transcription PCR (RT-PCR) and T cloning sequencing to evaluate their effects on pre-mRNA splicing.

**Results:**

Total 23 pathogenic variants including 11 novel variants in *NTRK1* were identified in 18 unrelated families with CIPA. Functional assays confirmed that five of these variants disrupted the normal splicing of *NTRK1*, resulting in multiple aberrant splicing patterns, including two exon-skipping events (c.428 + 273A>T, c.850 + 5G>A), three intron retentions (c.2187 + 389C>T, c.2188–459G>T, c.287 + 4A>C), and one pseudoexon insertion (c.2188–459G>T).

**Conclusion:**

This study expands the spectrum of pathogenic variants in *NTRK1* and improves the genetic diagnosis of CIPA. The functional characterization of five novel non-canonical splicing variants provides deeper insight into the molecular pathogenesis of this disorder and establishes a foundation for future precision medicine approaches in CIPA.

## 1 Introduction

Congenital insensitivity to pain with anhidrosis (CIPA; MIM 256800), also classified as hereditary sensory and autonomic neuropathy type IV (HSAN IV) (Axelrod and Gold-von Simson, 2007), is a rare autosomal recessive disorder with an estimated prevalence of one in 600,000 to 950,000 in Japan (Haga et al., 2013). The core clinical manifestations are present from birth and include recurrent episodes of unexplained fever, anhidrosis (absence of sweating), and insensitivity to noxious stimuli. As development progresses, affected individuals gradually exhibit delayed intellectual development and self-mutilating behavior (Indo et al., 1996).

Genetically, CIPA is caused by biallelic pathogenic variants in the neurotrophic receptor tyrosine kinase 1 (*NTRK1*) gene located on chromosome 1q23.1. *NTRK1*, the first identified member of the *NTRK* oncogene family (Martin-Zanca et al., 1986), encodes two functionally similar isoforms of TrkA that differ in tissue distribution: the longer isoform (796 amino acids [aa]) is neuron-specific, whereas the shorter isoform (790 aa) is expressed in non-neuronal cells. Structurally, TrkA is a transmembrane receptor composed of an extracellular domain, a single-pass transmembrane domain, and an intracellular domain that includes the kinase region and a C-terminal tail (Barbacid, 1995). As a high-affinity receptor for nerve growth factor (NGF), TrkA is highly expressed in NGF-dependent sensory and sympathetic neurons within the peripheral nervous system (Klein et al., 1991). The binding of NGF to TrkA induces receptor dimerization, autophosphorylation, and activation of the downstream signaling pathways mitogen-activated protein kinase (MAPK), phosphatidylinositol 3-kinase-protein kinase B (PI3K-AKT), and phospholipase C gamma (PLCγ), which collectively promote neuronal survival, axonal growth, and pain signal transmission (Huang and Reichardt, 2001). Pathogenic variants in *NTRK1* abolish TrkA kinase activity and disrupt this critical neurotrophic signaling cascade (Moraes et al., 2022). This disruption leads to two major developmental defects in patients with CIPA: the loss of nociceptive neurons in dorsal root ganglia, which causes congenital pain insensitivity, and the failure of sympathetic nerves to innervate sweat glands, which results in anhidrosis (Indo, 2018).

More than 100 disease-associated variants of *NTRK1* have been recorded in the Human Gene Mutation Database (HGMD), including 31 pathogenic variants originally characterized in our prior studies (Geng et al., 2018; Zhao et al., 2020; Li et al., 2024). These variants encompass multiple types, including missense, nonsense, splicing, small deletions/insertions/indels, gross deletions, uniparental disomy, and complex structural rearrangements. Among these, atypical splicing variants can cause aberrant mRNA splicing by creating new splicing sites or activating cryptic splice sites. We previously reported several atypical splicing variants in *NTRK1* from CIPA patients. For example, the variant c.575–19G>A near the canonical GT-AG splice site results in a 17-bp retention in intron 5 (Geng et al., 2018); the variant c.287 + 5G>C leads to a 59-bp retention in intron 2 (Li et al., 2024); and the deep intronic variant c.2187 + 459G>A induces aberrant retention of a 459-bp segment at the 5’ end of intron 15 (Li et al., 2024). These findings highlight the critical contribution of non-coding regions to the molecular pathogenesis of CIPA.

In the present study, in order to identify pathogenic variants missed by conventional genetic testing, we performed whole-genome screening (WGS) in CIPA patients to screen for pathogenic intronic variants in *NTRK1*. We identified five novel pathogenic intronic variants, including two variants near canonical splice sites and three deep intronic variants. All variants were functionally validated to disrupt normal pre-mRNA splicing, and their distinct molecular consequences were characterized.

## 2 Materials and methods

### 2.1 Participants

A total of 18 Chinese Han families, each including a proband and both parents, were enrolled in this study based on the clinical characteristics of CIPA. The numbering system for CIPA families was consistent with that used in our prior studies (Geng et al., 2018; Zhao et al., 2020; Li et al., 2024). This research was approved by the Institutional Review Board of the Institute of Basic Medical Sciences, Chinese Academy of Medical Sciences, Beijing, China (Approval No. 015–2015). Written informed consent was obtained from all adult participants and from the legal guardians of participants under 18 years of age prior to enrollment for the documentation of clinical data and collection of peripheral blood samples.

### 2.2 DNA extraction

Peripheral venous blood samples (4 mL) were collected from probands and their parents into EDTA-anticoagulated tubes. Genomic DNA (gDNA) was extracted using the standard phenol–chloroform method. gDNA concentration and purity were assessed using a NanoPhotometer-N60 spectrophotometer (IMPLEN, Munich, Germany), and aliquots were stored at 4 °C for subsequent experiments.

### 2.3 Genetic testing

#### 2.3.1 PCR-sanger sequencing

PCR-Sanger sequencing was used to detect coding variants, validate candidate pathogenic variants identified by WGS, and determine the parental origin of each variant. First, PCR-based methods were applied to detect candidate pathogenic variants of *NTRK1* in the probands. PCR-Sanger sequencing was conducted to identify variants in coding regions, canonical splice sites, and the recurrent intronic variants c.851–33T>A and c.[851-798C>T; 851-794C>G] previously identified in Chinese CIPA cohorts (Geng et al., 2018; Zhao et al., 2020; Li et al., 2024). Gap-PCR and agarose gel electrophoresis were performed to detect potential gross deletions in *NTRK1* (Li et al., 2024). Probands with only a single identified heterozygous pathogenic *NTRK1* variant according to the above analyses were further analyzed using WGS for comprehensive variant detection (families CIPA-130 and CIPA-133).

#### 2.3.2 *In silico* analysis

The online tools NetGene2 (v2.42) and NNSplice (BDGP) were used to predict whether the identified intronic variants would create novel splice donor or acceptor sites and potentially disrupt pre-mRNA splicing. The web-based software Mutalyzer 2.0.35 was employed to predict aberrant proteins.

#### 2.3.3 RNA isolation, RT-PCR, and T cloning

T cloning combined with Sanger sequencing was performed to confirm the aberrant splicing patterns caused by the intronic variants. Briefly, total RNA was extracted from fresh peripheral blood using TRIzol, and cDNA was synthesized by RT-PCR (Cat No. RR047A, Takara Bio). Nested PCR was performed to amplify cDNA fragments covering at least one exon upstream and one exon downstream of each variant site (Supplementary Table S2). The purified products of the nested PCR were ligated into the PMD-19T vector (Cat No. 6013, Takara Bio) and transformed into *Escherichia coli* DH5α competent cells for amplification and T cloning. Because proband CIPA-138 was a compound heterozygote of a novel variant c.850 + 5G>A and a known variant c.851–33T>A (both located in intron 7) (Conr et al., 2022), samples from his mother were used to validate the pathogenicity of the variant c.850 + 5G>A.

## 3 Results

### 3.1 Clinical characteristics and variant spectrum

**FIGURE 1:**
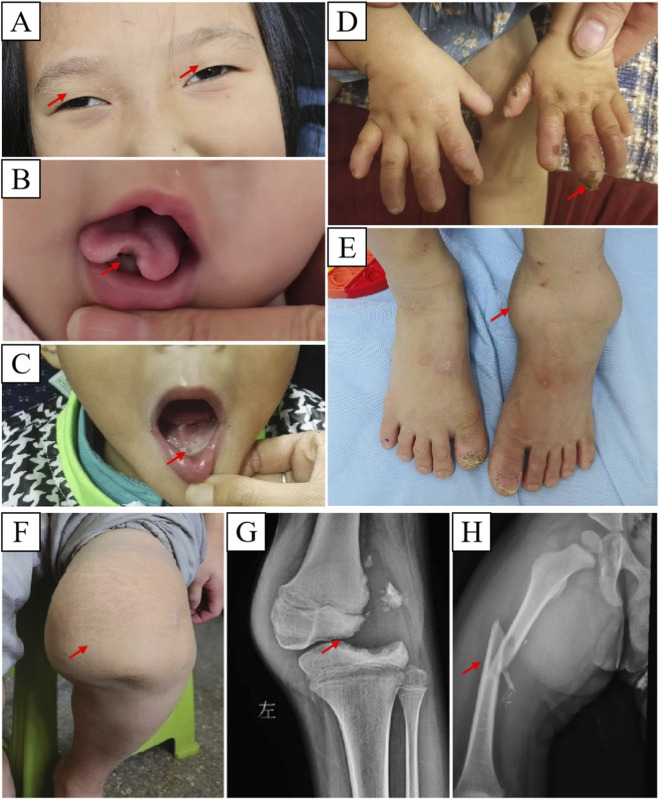
Typical clinical characteristics of the CIPA patients in this study. **(A)** Blepharoptosis. **(B–E)** Self-mutilation, including oral injury (tongue biting, tooth loss), limb damage (loss of the distal phalanx on fingers and toes), and skin ulceration. **(E–G)** Charcot joint. **(H)** Femoral fracture.

The cohort comprised 18 CIPA probands (6 females, 12 males) with a mean age of onset of 40 days. All probands exhibited the core clinical triad of CIPA: pain insensitivity, anhidrosis, and recurrent fever. As detailed in Table 1, common manifestations observed in all patients were fractures, developmental delay, and self-mutilation behavior. One proband presented with blepharoptosis (Figure 1A). Self-mutilation behaviors included tongue biting with resultant blunting or loss of the tongue tip (Figure 1B), tooth loss (Figure 1C), fingertip defects caused by biting (Figure 1D), and skin ulceration with scarring secondary to scratching (Figure 1E). Probands with severe bone diseases exhibited Charcot joints involving the ankles (Figure 1E) and knees (Figure 1F). Radiographic findings confirmed Charcot joint lesions, including osteoarthritis characterized by medial joint space narrowing and subchondral sclerosis and hyperplasia, as well as osteophyte formation at the margins of the tibial plateau and femoral condyles (Figure 1G). Recurrent fractures occurred in one-third of the probands (6 of 18) (Figure 1H).

**TABLE 1:** Clinical presentations of the 18 included patients.

Patient	Sex	Age at first visit	Siblings, no.	Recurrent fever	Insensitivity to pain	Anhidrosis	Number of fractures	Age at first fracture	Other bone diseases	Self-mutilation	Irascibility	Delayed gross motor development	Mental retardation	Blepharoptosis
CIPA-128	M	5 years	2	Y	Y	Y	2	2 weeks	Osteomyelitis	Y	N	N/A	N	Y
CIPA-129	M	15 years	1	Y	Y	Y	1	10 years	Charcot joint	Y	Y	Y	N	N
CIPA-130	F	3 years	2	Y	Y	Y	2	3 years 1 month	Osteomyelitis, charcot joint	Y	Y	Y	N	N
CIPA-131	F	2 years	0	Y	Y	Y	0	—	—	Y	Y	N/A	Y	N
CIPA-132	M	2 years	1	Y	Y	Y	1	2 years 6 months	—	Y	Y	N/A	Y	N
CIPA-133	M	6 months	0	Y	Y	Y	0	—	—	N	N	N/A	N	N
CIPA-134	M	1 year	0	Y	Y	Y	0	—	—	Y	N	Y	Y	N
CIPA-135	M	12 years	2	Y	Y	Y	2	9 years	Charcot joint	Y	N	Y	Y	N
CIPA-136	F	11 years	0	Y	Y	Y	0	—	—	N	N	Y	N	N
CIPA-137	M	11 years	0	Y	Y	Y	0	—	Osteomyelitis, charcot joint	Y	N	Y	Y	N
CIPA-138	M	9 years	6	Y	Y	Y	6	7 years	Genu valgus	Y	N	Y	Y	N
CIPA-139	M	4 years	1	Y	Y	Y	1	3 years 9 months	Osteomyelitis	Y	Y	Y	Y	N
CIPA-140	F	11 years	1	Y	Y	Y	1	4 years	Hip joint dislocation	Y	Y	Y	Y	N
CIPA-141	F	4 years	2	Y	Y	Y	2	2 years	Charcot joint	Y	Y	Y	Y	N
CIPA-142	M	1 year	1	Y	Y	Y	1	1 year	—	Y	Y	N	Y	N
CIPA-143	M	11 years	0	Y	Y	Y	0	—	—	Y	Y	Y	Y	N
CIPA-144	M	10 years	1	Y	Y	Y	1	3 years	Charcot joint	Y	Y	N	Y	N
CIPA-145	F	12 years	3	Y	Y	Y	3	8 years	Charcot joint	Y	Y	N	Y	N

F, female; M, male; N, no; N/A, not available; Y, yes.

The genotypic distribution of *NTRK1* variants is summarized in Table 2. We identified 23 distinct pathogenic variants in *NTRK1*, including 11 novel variants. Among these novel variants, four were non-canonical splicing variants, four were missense variants, two was a nonsense variant, and one was a small deletion. All CIPA probands harbored either homozygous or compound heterozygous *NTRK1* variants, consistent with the autosomal recessive inheritance pattern of CIPA.

**TABLE 2:** Pathogenic variants of *NTRK1* in the 18 patients with CIPA.

Patient	Zygote type	Allele origin	Variant location	Nucleotide (amino acid) change	Novel
CIPA-128	C-het	P	exon 5–7	c.429–230_851-756del	-
		M	exon 1	c.1A>G(p.Met1?)	-
CIPA-129	C-het	P	intron 7	c.851–33T>A	-
		M	exon 5	c.472T>G(p.Trp158Gly)	+
CIPA-130	C-het	P	intron 15	c.2187 + 389C>T	+
		M	exon 15	c.2056C>A(p.Arg686Ser)	+
CIPA-131	Hom	P	intron 7	c.851–33T>A	-
		M	intron 7	c.851–33T>A	-
CIPA-132	C-het	P	intron 7	c.[851-798C>T; 851-794C>G]	-
		M	intron 7	c.851–33T>A	-
CIPA-133	C-het	P	intron 5	c.574 + 1G>A	-
		M	intron 4; 15	c.[428 + 273A>T; 2188–459G>T]	+
CIPA-134	C-het	P	exon 8	c.963delG(p.Leu322Serfs*142)	-
		M	intron 2	c.287 + 4A>C	+
CIPA-135	C-het	P	exon 16	c.2293C>T(p.Arg765Cys)	-
		M	exon 13	c.1770C>A(p.Asp590Glu)	+
CIPA-136	C-het	P	exon 14	c.1915C>A(p.His639Asn)	+
		M	exon 6	c.646C>T(p.Gln216*)	-
CIPA-137	C-het	P	intron 7	c.851–33T>A	-
		M	intron 7	c.[851-798C>T; 851-794C>G]	-
CIPA-138	C-het	P	intron 7	c.851–33T>A	-
		M	intron 7	c.850 + 5G>A	+
CIPA-139	C-het	P	exon 2	c.238C>T(p.Gln80*)	+
		M	exon 13	c.1772_1785del(p.Leu591Profs*6)	+
CIPA-140	C-het	P	intron 7	c.851–33T>A	-
		M	exon 6	c.646C>T(p.Gln216*)	-
CIPA-141	Hom	P	intron 2	c.287+2dupT	-
		M	intron 2	c.287+2dupT	-
CIPA-142	C-het	P	exon 15	c.2060G>A(p.Trp687*)	+
		M	intron 7	c.[851-798C>T; 851-794C>G]	-
CIPA-143	C-het	P	intron 7	c.851–33T>A	-
		M	exon 8	c.963delG(p.Leu322Serfs*142)	-
CIPA-144	Hom	P	intron 7	c.851–33T>A	-
		M	intron 7	c.851–33T>A	-
CIPA-145	C-het	P	intron 5	c.575–19G>A(p.Pro194Leufs*9)	-
		M	exon 13	c.1786C>T(p.R596*)	-

C-het, compound heterozygote; Hom, homozygote; P, paternal; M, maternal; +, novel variant; -, known variant.

### 3.2 Functional assay of non-canonical splicing variants

#### 3.2.1 Splicing effects prediction

The impacts of the identified variants on pre-mRNA splicing were predicted using NetGene2 and NNSplice with default thresholds. NetGene2 analysis revealed that variants c.2187 + 389C>T, c.2188–459G>T, c.287 + 4A>C, and c.850 + 5G>A resulted in a 50% reduction in the score of the corresponding donor sites (Supplementary Table S1), suggesting disruption of the canonical splicing. NNSplice predictions indicated that c.2188–459G>T (|Δscore| = 0.28 > 0.2) may moderately alter splicing. c.287 + 4A>C (|Δscore| = 0.96 > 0.8) and c.850 + 5G>A (|Δscore| = 0.87 > 0.8) were highly likely to cause aberrant splicing. SpliceAI predicted the emergence of a new donor splice site near the variant c.2188–459G>T (score 0.82), and the loss of the original donor site after the variant c.287 + 4A>C (score −0.38) and c.850 + 5G>A (score −0.98) (Table 3).

**TABLE 3:** *In silico* splicing alterations of five novel *NTRK1* variants.

Variant	NetGene2 (donor site Δscore)	NNSplice (donor site Δscore)	SpliceAI (donor site Δscore)
c.2187 + 389C>T	−0.35	0	−0.05
c.428 + 273A>T	0.03	0	0
c.2188–459G>T	0.34	0.28	0.82
c.287 + 4A>C	−0.71	−0.96	−0.38
c.850 + 5G>A	−0.91	−0.87	−0.98

#### 3.2.2 Exon skipping

**FIGURE 2:**
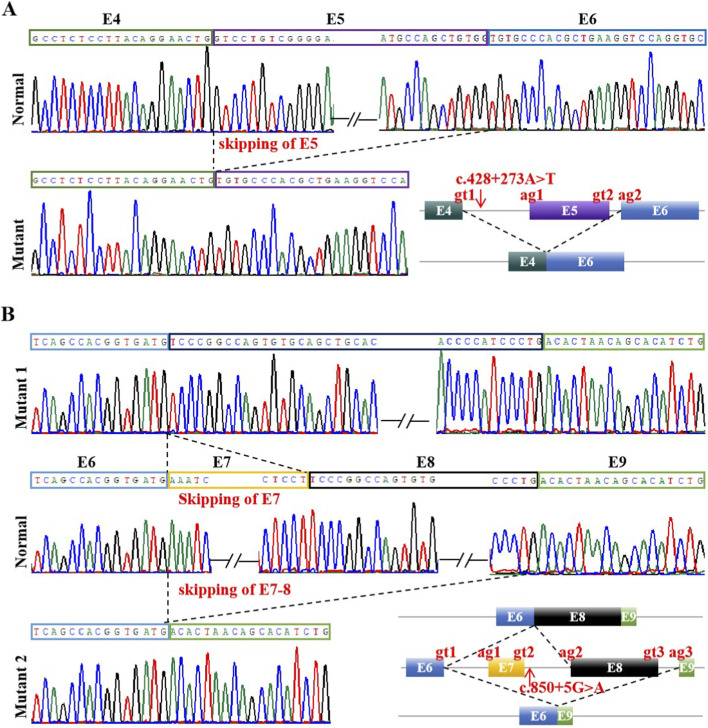
Intronic variants associated with exon skipping. **(A)** The sequencing results and schematic representation of aberrant splicing pattern induced by variant c.428 + 273A>T; **(B)** The sequencing results and schematic representation of aberrant splicing patterns induced by variant c.850 + 5G>A.

T cloning-based Sanger sequencing demonstrated that the deep intronic variant c.428 + 273A>T in intron four of *NTRK1* resulted in the skipping of the entire exon 5, which account for 13.3% (2/15) (Figure 2A). The variant c.850 + 5G>A in intron 7 produced two aberrant splicing patterns: sole skipping of exon 7, which account for 20% (2/10), and concurrent skipping of exons 7 and 8, with the proportion of 20% (1/10) (Figure 2B).

#### 3.2.3 Intronic retention and pseudoexon incorporation

**FIGURE 3:**
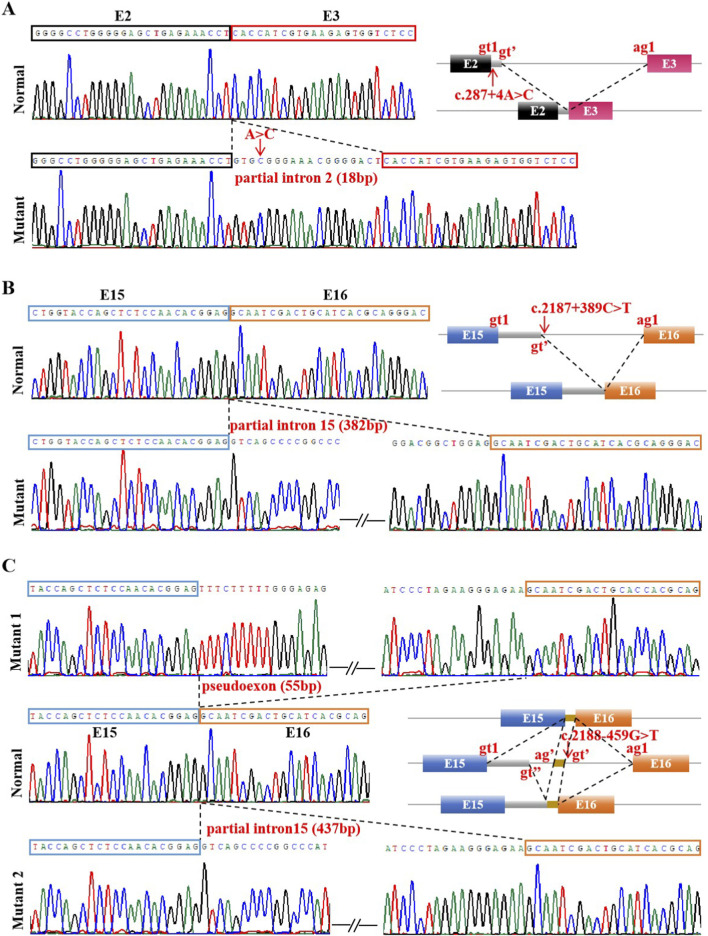
Intronic variants associated with intron retention or pseudoexon inclusion. **(A)** The sequencing results and schematic representation of aberrant splicing pattern induced by variant c.287+4A>C; **(B)** The sequencing results and schematic representation of aberrant splicing pattern induced by variant c.2187 + 389C>T; **(C)** The sequencing results and schematic representation of aberrant splicing pattern induced by variant c.2188‐459G>T.

The variant c.287 + 4A>C in intron 2 of *NTRK1* aberrantly activates a cryptic 5′ splice donor site (GT) located 15–16 nucleotides downstream, resulting in the abnormal inclusion of an 18-bp intronic sequence at the 5′ terminus of intron 2, with the proportion of 50% (6/12) (Figure 3A). The deep intronic variant c.2187 + 389C>T activates a cryptic 5′ splice donor site (GT) located 5-6 nucleotides upstream, leading to the inclusion of a 382-bp fragment at the 5′ terminus of intron 15, with the proportion of 21.4% (3/14) (Figure 3B). The deep intronic variant c.2188–459G>T induces two distinct aberrant splicing events: (i) the inclusion of a 55-bp pseudoexon derived from the middle region of intron 15, with the proportion of 12.5% (2/16); and (ii) the pathological retention of a 437-bp intronic segment spanning the 382-bp 5′-terminal sequence and the internal 55-bp pseudoexon sequence within intron 15,with the proportion of 6.25% (1/16) (Figure 3C).

#### 3.2.4 Protein prediction

The protein-level consequences of aberrant splicing variants were predicted using the online tool Mutalyzer (Table 4). Exon-skipping events that disrupt the open reading frame (i.e., when the length of the skipped exon is not a multiple of three) induce frameshifts, generating premature termination codons (PTCs) and yielding C-terminally truncated proteins. The exon 5-skipping event induced by variant c.428 + 273A>T results in a 146-bp deletion. This alteration is predicted to cause a valine-to-cysteine substitution at codon 144 and introduce a PTC 65 residues downstream (p.Val144Cysfs*65), leading to a truncated protein product. Variant c.850 + 5G>A generates two distinct aberrant splicing isoforms via exon-skipping events. The transcript lacking exon 7 carries a 133-bp deletion, which is predicted to cause a lysine-to-serine substitution at codon 240 and introduce a PTC 180 residues downstream (p.Lys240Serfs*180), yielding a truncated protein. Alternatively, the transcript with skipping of exons 7 and 8 exhibits a 460-bp deletion, which is predicted to result in a lysine-to-threonine substitution and generate a PTC 71 residues downstream (p.Lys240Thrfs*71), producing a shorter polypeptide.

**TABLE 4:** Predicted proteins derived from aberrant transcripts.

Variants	Aberrant transcripts	Prediction
c.287 + 4A>C	c.287 + 1_287 + 18ins	p.Thr97*
c.428 + 273A>T	c.429_574del	p.V144Cfs*65
c.850 + 5G>A	c.718_850del	p.K240Sfs*180
	c.718_1177del	p.K240Tfs*71
c.2187 + 389C>T	c.2188 + 1_2188 + 382ins	p.A730Vfs*69
c.2188–459G>T	c.2188–519_2188-465ins	p.A730Vfs*69
	(c.2187 + 1_2187 + 382) + (c.2188–519_2188–465) ins	p.A730Ffs*28

Intron retention events that insert non-triplet nucleotide sequences typically cause frameshift-mediated nonsense mutations, leading to a PTC and protein truncation. Variants c.2187 + 389C>T and c.2188–459G>T cause a 382-bp intron retention and a 55-bp pseudoexon inclusion, respectively. Based on the mutated transcript, the encoded protein is predicted to undergo an alanine-to-valine change at position 730, resulting in a stop codon 69 residues downstream (p.Ala730Valfs*69) and leading to an elongated polypeptide. Variant c.2188–459G>T also induces an aberrant transcript retaining a 437-bp segment of intron 15, which is predicted to cause an alanine-to-phenylalanine substitution at position 730 and introduce a PTC 28 residues downstream (p.Ala730Phefs*28), resulting in a truncated protein. Variant c.287 + 4A>C causes aberrant splicing with an 18-bp retention at the 5′ splice site of intron 2, which is predicted to introduce a PTC at position 97 (p.Thr97*), leading to premature translational termination and a C-terminally truncated protein product.

### 3.3 Pathogenicity analysis of *NTRK1*

In accordance with the ACMG/AMP Variant Classification Guidelines, we evaluated the evidence of pathogenicity to classify the 11 novel variants (Table 5). Loss-of-function (LoF) variants were assigned very strong evidence of pathogenicity (PVS1). The confirmed absence of these novel variants in major large-scale population databases, including ExAC, 1,000 Genomes, and gnomAD, constituted moderate evidence of pathogenicity (PM2). Detection of the variants in trans at the *NTRK1* provided additional moderate evidence (PM3). Furthermore, the rarity of benign missense variants and their established role as a disease-causing mechanism in *NTRK1* fulfill the PP2 criterion. Alongside a highly specific disease phenotype (PP4), both provide supporting evidence of pathogenicity. Crucially, our functional studies utilizing RT-PCR and T cloning from patient-derived samples provided *in vitro* evidence that all five non-canonical variants directly result in aberrant pre-mRNA splicing. This functional validation firmly satisfies the criteria for strong evidence of pathogenicity (PS3).

**TABLE 5:** Variant pathogenicity classification following the ACMG/AMP guidelines.

Variant	Pathogenic evidence	Classification
c.238C>T(p.Gln80*)	PVS1+PM2+PM3+PP4	P
c.1772_1785del	PVS1+PM2+PM3+PP4	P
c.2060G>A(p.Trp687*)	PVS1+PM2+PM3+PP4	P
c.472T>G(p.Trp158Gly)	PM2+PM3+PP2+PP4	LP
c.2056C>A(p.Arg686Ser)	PM2+PM3+PP2+PP4	LP
c.1770C>A(p.Asp590Glu)	PM2+PM3+PP2+PP4	LP
c.1915C>A(p.His639Asn)	PM2+PM3+PP2+PP4	LP
c.2187 + 389C>T	PM2+PM3+PP4 (+PP3+PS3)	VUS→P
c.[428 + 273A>T; 2188–459G>T]	PM2+PM3+PP4 (+PP3+PS3)	VUS→P
c.287 + 4A>C	PM2+PM3+PP4 (+PP3+PS3)	VUS→P
c.850 + 5G>A	PM2+PM3+PP4 (+PP3+PS3)	VUS→P

P, pathogenic; LP, likely pathogenic; VUS, variant of uncertain significance.

We confirmed that variants c.2187 + 389C>T, c.2188–459G>T, c.287 + 4A>C, and c.850 + 5G>A are predicted *in silico* to affect splicing, meeting supporting evidence PP3. Additionally, functional studies verified that all five of these non-canonical splicing variants cause aberrant splicing, providing strong evidence of pathogenicity (PS3). With the addition of these new lines of pathogenic evidence, the classification of variants c.2187 + 389C>T, c.428 + 273A>T, c.2188–459G>T, c.287 + 4A>C and c.850 + 5G>A was upgraded from variants of uncertain significance (VUS) to pathogenic variants.

## 4 Discussion

In the present cohort, all patients exhibited the classic clinical triad of CIPA: congenital pain insensitivity, anhidrosis, and recurrent hyperpyrexia. Beyond these pathognomonic features, a substantial proportion developed severe complications. Skeletal manifestations were particularly prominent: 66.7% of the patients (12 of 18) had a history of fractures, and 61.1% (11 of 18) progressed to disabling osteopathies, including osteomyelitis and Charcot joints. These findings underscore the critical importance of orthopedic surveillance and early intervention in the clinical management and complication prevention of CIPA.

Regarding neurological manifestations, self-mutilation was the most frequent feature, affecting 94.4% (17 of 18) of the cohort. This incidence is consistent with the 88.2% incidence (112 of 127) previously found in large-scale studies (Geng et al., 2018; Zhao et al., 2020; Li et al., 2024). Furthermore, most patients demonstrated neurodevelopmental and psychiatric impairments, including gross motor delay (61.1%, 11 of 18), irritability (61.1%, 11 of 18), and intellectual disability (72.2%, 13 of 18). Notably, all patients showed severe deficits in arithmetic ability and logical reasoning, further supporting the pivotal role of the *NTRK1* signaling pathway in higher-order neurocognitive development.

Blepharoptosis has been documented in our previous studies (Geng et al., 2018), and we identified one case in the present cohort. This observation suggests that blepharoptosis represents a rare but specific phenotypic feature of CIPA and may serve as an auxiliary clinical diagnosis.

In this study, we identified 23 pathogenic *NTRK1* variants, including 11 novel missense, nonsense, and splicing variants. In particular, we characterized five novel non-canonical pathogenic splicing variants. The variant c.287 + 4A>C was found to induce retention of intron 2. The resulting aberrant transcript is predicted to generate a truncated protein, likely impairing ligand-binding specificity (Conr et al., 2022). The variant c.850 + 5G>A produced complex alternative splicing patterns, including two distinct exon-skipping events: skipping of exon 7 alone, or concurrent skipping of exons 7 and 8. Loss of exon 7 is expected to disrupt the immunoglobulin (Ig)-like domain of TrkA (a critical region for NGF binding and receptor dimerization), compromising ligand–receptor interactions. Furthermore, the combined skipping of exons 7 and 8 disrupts the Ig-like domain and causes loss of the transmembrane domain (Bedanokova et al., 2024).

Notably, Sun et al. previously investigated the effect of c.850 + 5G>A on *NTRK1* splicing using a minigene system and reported two aberrant transcripts with 13-bp and 25-bp deletions at the 3′end of exon 7 (Sun et al., 2024). In contrast, our *in vivo* analysis using RT-PCR and T cloning sequencing revealed complete skipping of exon 7 or exons 7 and 8. This discrepancy highlights the inherent limitations of minigene-based splicing assays. Although a minigene system can detect splicing alterations, physiological splicing depends on the full genomic context and the coordinated function of multiple spliceosomal components, which may not be fully recapitulated *in vitro*. We therefore propose that transcriptomic analysis using endogenous RNA provides a more biologically relevant evaluation of splicing outcomes.

In addition, we identified a complex allele harboring two cis-acting deep intronic variants: c.[428 + 273A>T; 2188–459G>T]. Functional assays confirmed that both variants independently cause aberrant splicing. Specifically, c.428 + 273A>T leads to skipping of exon 5, which disrupts the leucine-rich repeat motif in the extracellular domain. This structural change is predicted to reduce the binding affinity between TrkA and NGF (Indo, 2002). Moreover, variants c.2187 + 389C>T and c.2188–459G>T result in partial retention of intron 15, which is predicted to cause a C-terminal extension of the tyrosine kinase domain (Franco et al., 2020). The structural alterations and underlying pathogenic mechanisms of the proteins encoded by these aberrant transcripts remain to be elucidated through future experimental validation of protein expression and functional signaling pathways.

However, because these two variants reside in cis on the same chromosome (c.[428 + 273A>T; 2188–459G>T]), their combined, synergistic effect on *NTRK1* pre-mRNA processing within the actual patient context may be considerably more intricate than what is observed in isolated assays. In future investigations, long-range RT-PCR coupled with Sanger sequencing could be deployed to definitively map the full-length transcript configurations generated by this complex allele. It is worth noting, however, that since both the individual variants and their combination ultimately lead to a severe disruption of the *NTRK1* gene function, the clinical pathogenicity and phenotypic impact on the patient are expected to remain essentially identical regardless of the underlying transcript complexity.

In this study, we evaluated the functional impacts of novel non-canonical splicing variants using patient-derived peripheral blood RNA. However, interpreting the relative proportions of observed transcripts requires caution, particularly in compound heterozygous individuals. For several probands in our cohort, the splicing variant was paired with a coding variant on the opposite allele. Under such conditions, the transcript composition in the total cDNA pool does not strictly reflect the intrinsic, isolated splicing efficiency of the splicing-variant allele itself. Confounding factors, such as nonsense-mediated mRNA decay (NMD) targeting transcripts with premature termination codons (PTCs), or differential baseline transcriptional activity between the maternal and paternal alleles, can significantly alter the relative abundance of functional versus aberrant isoforms. Therefore, the transcript ratios characterized via RT-PCR and TA cloning represent the steady-state mRNA equilibrium in peripheral blood rather than absolute, single-allele splicing kinetics. Consequently, these transcript proportions should be interpreted with prudence when drawing correlations regarding splicing severity or clinical pathogenicity.

In this cohort, splicing variants accounted for approximately 58% of all pathogenic alleles, confirming their role as the predominant molecular mechanism underlying CIPA. Therefore, the identification of non-canonical splicing variants is critical for improving diagnostic yield in patients with clinically suspected CIPA. Our findings support the use of WGS as an optimal strategy for CIPA patients in whom no pathogenic variants are detected by whole-exome sequencing.

Five variants in the introns of *NTRK1* were found using WGS, but only three of them were predicted (NetGene2, NNSplice and SpliceAI) to affect splicing. In silico analysis predicted that the variant c.2188–459G>T might create a novel splice donor site. Functional assays subsequently revealed that this variant generated a novel splice donor site (gt’) at position −463 (upstream of the mutation site) and a novel splice acceptor site (ag’) 55 bp further upstream, leading to the inclusion of a 55-bp pseudoexon. An alternative aberrant transcript generated an additional novel splice donor site (gt’’) at position +382, resulting in the partial retention of the 5′ end of the intron. For the variant c.287 + 4A>C, predictions suggested the loss of the upstream canonical splice donor site; experimental results demonstrated the activation of a cryptic splice donor site (gt’) at position +18, causing an 18-bp intron retention. Furthermore, the variant c.850 + 5G>A was predicted to abolish its upstream splice donor site, which functionally resulted in either the isolated skipping of exon 7 (loss of ag1 and gt2) or the concurrent skipping of both exons 7 and 8 (loss of ag1, gt2, ag2 and gt3). Overall, the *in silico* splicing predictions were largely consistent with the experimentally observed transcripts for several variants. However, notable discrepancies were observed; for instance, the splicing impacts of variants c.428 + 273A>T and c.2187 + 389C>T were underestimated by SpliceAI. Although highly sensitive for screening, deep learning algorithms lack mechanistic interpretability, which can be complemented by motif-level tools like MaxEntScan. Crucially, our functional analyses confirmed that all five variants impaired or altered the recognition of original splice sites, generating abnormal transcripts. This highlights that while computational predictions are valuable for initial screening, functional assays remain indispensable for definitively characterizing complex deep intronic variants.

In our diagnostic pipeline, conventional PCR and Sanger sequencing covering all exons and canonical splice sites of *NTRK1* were initially performed for all probands. For patients in whom only a single pathogenic allele was identified, we employed WGS to uncover the second variant. We opted for WGS over targeted *NTRK1* sequencing or direct RNA sequencing for several critical reasons. First, RNA-based approaches heavily depend on the availability and stability of target transcripts in accessible tissues like peripheral blood. This can be technically challenging due to tissue-specific expression differences or the rapid degradation of mutant transcripts via nonsense-mediated mRNA decay (NMD). Second, compared to targeted gene panels, WGS provides a comprehensive assessment of the genomic landscape. It not only enables the precise identification of deep intronic splicing variants but also facilitates the concurrent detection of complex structural variants, large genomic deletions, and regulatory region alterations that conventional targeted approaches might overlook. Therefore, integrating WGS into the diagnostic method serves as a highly effective and optimal strategy for resolving genetically undiagnosed CIPA cases.

## 5 Conclusion

The present study has expanded the pathogenic variant spectrum of *NTRK1* and improved the genetic diagnostic rate for CIPA by identifying 11 novel variants. We provide evidence for bioinformatics analysis while compensating for the deficiencies of these tools in assessing the pathogenicity of non-canonical splicing through the combined use of bioinformatics tools and *in vitro* functional experiments. Overall, our study presents a comprehensive and accurate guidance framework for the diagnosis of pathogenic gene variations in undiagnosed cases of CIPA, thereby facilitating the precise diagnosis of the condition.

## Data Availability

The raw data and research materials related to the experiment can be obtained from the corresponding authors upon reasonable request.
