# Supplementary material for: Novel deep intronic variants in NTRK1 underlying congenital insensitivity to pain with anhidrosis
Source: Front Genet. 2026 Jul 2;17:1852317. doi: 10.3389/fgene.2026.1852317 (PMC13372274; doi:10.3389/fgene.2026.1852317)
Supplement: Supplementary file 1 [file Table1.DOCX]

Table S1: Pathogenicity predicted by In Silico

| Variant | Score from NetGene2 | | |  | Score from NNSplice | | |
| --- | --- | --- | --- | --- | --- | --- | --- |
|  | Wild-type donor site | Mutant donor site | Δscore |  | Wild-type donor site | Mutant donor site | Δscore |
| c.2187+389C>T | 0.35 | 0 | -0.35 |  | 0 | 0 | 0 |
| c.428+273A>T | 0.66 | 0.7 | 0.03 |  | 0 | 0 | 0 |
| c.2188-459G>T | 0.45 | 0.79 | 0.34 |  | 0.71 | 0.99 | 0.28 |
| c.287+4A>C | 0.71 | 0 | -0.71 |  | 0.96 | 0 | -0.96 |
| c.850+5G>A | 0.91 | 0 | -0.91 |  | 0.87 | 0 | -0.87 |

Table S2: *NTRK1* RT-nested PCR primers used in this study

| Variant | Primer | Sequence (5’-3’) | Tm (℃) |
| --- | --- | --- | --- |
| c.287+4A>C | 1st-F | CTGCTGGCTTGGCTGATACT | 58 |
|  | 1st-R | CACTGCAGCTTCTGTTCAGG |  |
|  | 2nd-F | AGAGAACCTGACTGAGCT | 58 |
|  | 2nd-R | CAAGAACAGTGCAGAGGGTT |  |
| c.428+273A>T | 1st-F | CTGGCTTGGCTGATACTGG | 58 |
|  | 1st-R | GGAGACGTTGACCTGAACAG |  |
|  | 2nd-F | AGAGAACCTGACTGAGCTCT | 58 |
|  | 2nd-R | CGTTGACCTGAACAGAGACC |  |
| c.850+5G>A | 1st-F | AACCCTCTGCACTGTTCTTG | 58 |
|  | 1st-R | TGCCACCCAATGTCATGAAA |  |
|  | 2nd-F | AACAGAAGCTGCAGTGTCAT | 58 |
|  | 2nd-R | GTAGAAAGGAAGAGGCAGGC |  |
| c.2187+389C>T  c.2188-459G>T | 1st-F | CCCCTGCTCATGGTCTTTG | 58 |
|  | 1st-R | AACATCCTCTACTCCCAGCC |  |
|  | 2nd-F | GGTCTTTGAGTATATGCGGCA | 58 |
|  | 2nd-R | ATTGCTATGACGGGACCTTG |  |
